# Embryonic Amoxicillin Exposure Has Limited Impact on Liver Development but Increases Susceptibility to NAFLD in Zebrafish Larvae

**DOI:** 10.3390/ijms25052744

**Published:** 2024-02-27

**Authors:** Peng Gao, Cheng Chang, Jieling Liang, Fen Du, Ruilin Zhang

**Affiliations:** 1 TaiKang Medical School (School of Basic Medical Sciences), Wuhan University, Wuhan 430071, China; 2020203010018@whu.edu.cn (P.G.); chaeng@whu.edu.cn (C.C.); 2021203010023@whu.edu.cn (J.L.); fen.du@whu.edu.cn (F.D.); 2 Hubei Provincial Key Laboratory of Developmentally Originated Disease, Wuhan 430071, China

**Keywords:** amoxicillin: clavulanic acid, developmental toxicity, disease susceptibility, NAFLD

## Abstract

Amoxicillin is commonly used in clinical settings to target bacterial infection and is frequently prescribed during pregnancy. Investigations into its developmental toxicity and effects on disease susceptibility are not comprehensive. Our present study examined the effects of embryonic amoxicillin exposure on liver development and function, especially the effects on susceptibility to non-alcoholic fatty liver disease (NAFLD) using zebrafish as an animal model. We discovered that embryonic amoxicillin exposure did not compromise liver development, nor did it induce liver toxicity. However, co-treatment of amoxicillin and clavulanic acid diminished BESP expression, caused bile stasis and induced liver toxicity. Embryonic amoxicillin exposure resulted in elevated expression of lipid synthesis genes and exacerbated hepatic steatosis in a fructose-induced NAFLD model, indicating embryonic amoxicillin exposure increased susceptibility to NAFLD in zebrafish larvae. In summary, this research broadens our understanding of the risks of amoxicillin usage during pregnancy and provides evidence for the impact of embryonic amoxicillin exposure on disease susceptibility in offspring.

## 1. Introduction

Environmental exposures during critical developmental periods can influence tissue development and interactions, leading to long-lasting effects on health [1,2]. The fetus during the prenatal stage is vulnerable to many environment factors including maternal diseases, lifestyle, and exposure to exogenous chemicals and drugs, which can influence fetal development as well as pathophysiology later in life [3,4,5]. The most prescribed medications during pregnancy are antibiotics [6]. Previous studies indicate that using antibiotics such as penicillin and piperacillin during pregnancy may reduce fetal birth weight, lead to neurological disorders, and cause atopic diseases after birth, including atopic dermatitis and asthma [7,8]. Pregnant women often encounter respiratory and urinary tract infections, and amoxicillin is the primary antibiotic used to treat such diseases. Although amoxicillin is well-known for not causing severe malformations, it is still unclear whether it is completely non-toxic to the fetus.

Amoxicillin is a broad-spectrum beta-lactam antibiotic of the penicillin class that has strong inhibitory and bactericidal effects on most pathogenic Gram-positive and Gram-negative bacteria [9,10] and thus is widely used in clinical settings [11,12,13]. In adverse drug reactions (ADR) caused by antibiotics, amoxicillin accounts for 1.5%, and the harm of ADR in infants and young children aged 0–14 is more severe [14]. Amoxicillin use during pregnancy is not associated with most congenital malformations [15]. However, studies have suggested that pregnant women exposed to amoxicillin in the first trimester are at an increased risk of having infants with oral clefts [15,16]. In clinical practice, amoxicillin and clavulanic acid are frequently paired to enhance antibacterial efficacy [9,17]. During early pregnancy, exposure to amoxicillin/clavulanic acid in the uterus poses a risk of severe malformations to the fetus [18]. Thus, it is critical to establish a corresponding model to explore the impact of amoxicillin exposure during embryonic development.

Non-alcoholic fatty liver disease (NAFLD) is a metabolic-related type of fatty liver, distinct from those linked to alcohol intake [19,20]. As obesity and its related complications become more prevalent worldwide, the incidence of NAFLD has been rising and now has a global prevalence rate between 14% and 32% [21]. Dietary NAFLD models include animals fed a high-fat diet (HFD), a methionine-choline deficient diet, or a fructose diet (FD) [22,23,24]. Previous research has shown that in mouse models, HFD alone can alter the composition of the gut microbiota [25], while using antibiotics can alter the ability of the microbiome to adapt to a HFD. The use of pulsed antibiotic treatment to mimic pediatric antibiotic use highlights the alterations in metabolic development caused by antibiotic-induced microbiota perturbations in early life [26]. Additionally, hepatic metabolism can be altered with early-life antibiotic exposure [27]. However, information regarding the impact of early antibiotic exposure on NAFLD is limited. It is of great interest to investigate the susceptibility to NAFLD after embryonic amoxicillin exposure.

Zebrafish serve as an excellent vertebrate animal model and are extensively employed in genetic and developmental biology studies. Additionally, they are frequently used as models for mechanistic studies of human diseases such as NAFLD, cardiomyopathy, and atherosclerosis [28,29,30,31]. Zebrafish have over 80% of human disease-related targets and multiple drug metabolic pathways, and the physiology of zebrafish is typically well-conserved in humans [32,33,34]. Compounds identified through zebrafish screenings have demonstrated comparable effects in both rodent models and humans [35,36]. Researchers expect that establishing metabolomics analysis and toxicity-related reporter lines will further expand the applications of zebrafish.

This study aimed to explore the potential hepatotoxicity of amoxicillin and disease susceptibility later in life that results from embryonic amoxicillin exposure using zebrafish. First, we demonstrated that amoxicillin treatment alone did not cause hepatotoxicity in zebrafish larvae. After co-treatment of amoxicillin and clavulanic acid, liver development and lipid metabolism were affected. Further experiments indicated that amoxicillin treatment during embryonic stages rendered zebrafish larvae more susceptible to diet-induced NAFLD. Overall, our findings establish a zebrafish model to investigate the impact of prenatal drug exposure on offspring and provides evidence for the impact of embryonic amoxicillin exposure on disease susceptibility in offspring.

## 2. Results

### 2.1. Amoxicillin Treatment Does Not Affect Development of Essential Organs in Zebrafish

**Figure 1:**
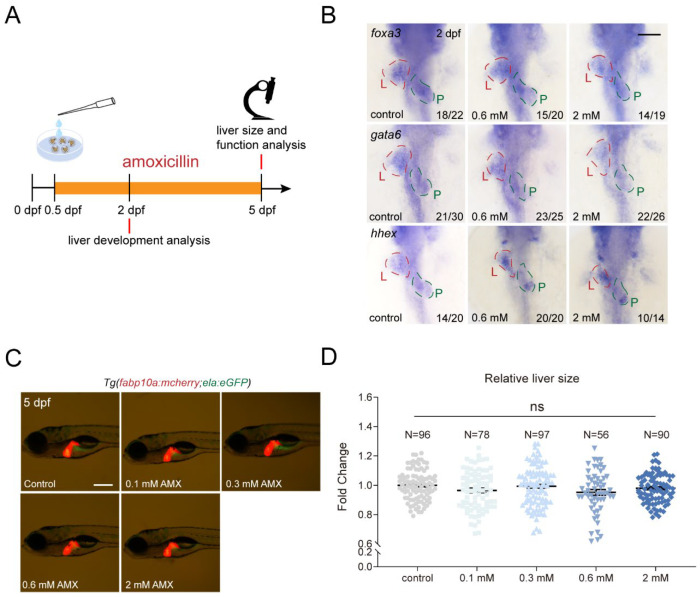
Amoxicillin treatment does not affect zebrafish liver development. (**A**) Schematic diagram of amoxicillin treatment. (**B**) Whole-mount in situ hybridization (WISH) revealed the expression of *foxa3*, *gata6* and *hhex* at 2 dpf after amoxicillin treatment. Ventral view, anterior to the top. L—liver (red dashed lines); P—pancreas (green dashed lines). Numbers indicate the ratio of representative staining observed. (**C**) Liver (red) size at 5 dpf after amoxicillin treatment as shown in the transgenic reporter line *Tg(fabp10a:mCherry; ela:eGFP)*. Lateral view, anterior to the left. (**D**) Quantification of relative liver size at 5 dpf. The numbers of larvae used for each condition are indicated. In the ANOVA analysis, ns—not significant. Scale bars, 100 µm. AMX—amoxicillin; dpf—days post-fertilization.

To determine whether amoxicillin treatment would impact zebrafish embryonic development, embryos were subjected to prolonged amoxicillin treatment with concentration gradients (0.1–2 mM) from 0.5 to 5 days post-fertilization (dpf), a scheme to prevent dramatic embryo malformations [37] that could arise from treatments before the gastrula stage (Figure 1A). 1-phenyl 2-thiourea (PTU) is typically added from 1 dpf onward to prevent pigmentation. However, for the consistency of drug treatment, we added PTU to the egg water at 12 h post-fertilization (hpf) when the treatment started. This resulted in a lower hatching rate of zebrafish embryos than usual at 48 hpf, but the hatching rate caught up at 60 hpf (Figure S1A,B). We also observed that increased concentrations of amoxicillin resulted in a decrease in larval body length at 4 dpf (Figure S1C,D), in agreement with previous reporting [38]. Through whole-mount in situ hybridization (WISH), we observed that the expressions of early liver differentiation markers *foax3*, *gata6*, and *hhex* remained unchanged at 2 dpf upon amoxicillin treatment (Figure 1B). At 5 dpf, the assessment of liver area using *Tg(fabp10a:mCherry; ela:eGFP)* report line revealed no significant reduction in liver area following amoxicillin treatment (Figure 1C,D). Given the pivotal role of the heart in early development, we also investigated cardiac development and function and ascertained that amoxicillin treatment had no discernible impact on ventricular end-diastolic volume, end-systolic volume, fractional shortening, and heart rate (Figure S2). These data suggest that amoxicillin treatment does not compromise the development of vital organs such as the liver and heart in zebrafish.

### 2.2. Amoxicillin Treatment Has Limited Impact on Zebrafish Liver Function

**Figure 2:**
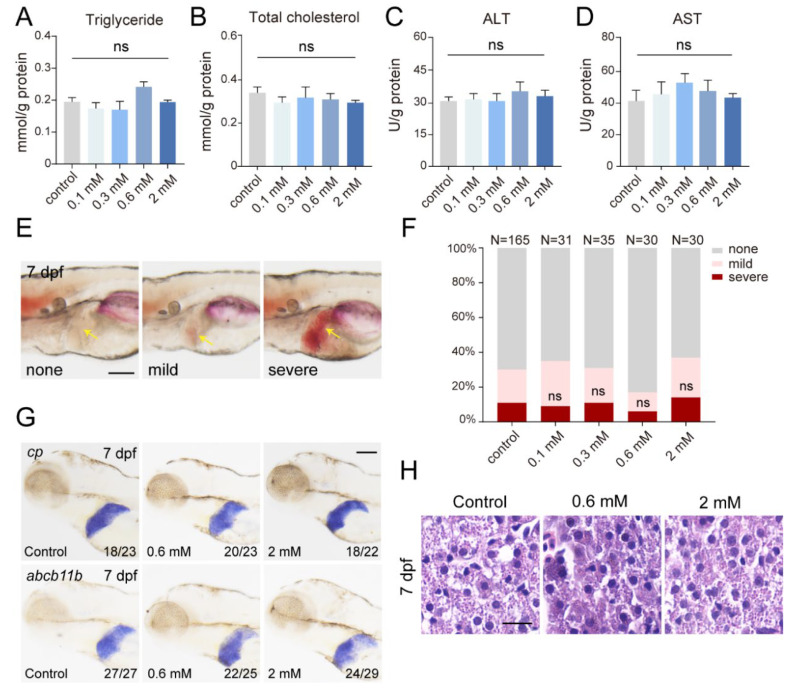
Amoxicillin treatment has limited impact on zebrafish liver function. (**A**–**D**) Quantification of triglyceride, total cholesterol, ALT activity, and AST activity in 5 dpf larvae after amoxicillin treatment from 0.5 to 5 dpf. Three independent experiments. Mean + s.e.m. In ANOVA analysis, ns—not significant. (**E**) Representative Oil red O [3] staining of larvae at 7 dpf. Larvae were categorized as having none, mild, or severe hepatic steatosis. Arrows point to the liver area. (**F**) Quantification of hepatic steatosis by ORO staining in 7 dpf larvae after amoxicillin treatment. The numbers of larvae analyzed in each group are indicated. In Chi-square tests, ns—not significant. (**G**) WISH revealed the expression of *cp* and *abcb11b* at 7 dpf after amoxicillin treatment. Numbers indicate the ratio of representative staining observed. (**H**) H&E staining of liver tissues at 7 dpf after amoxicillin treatment from 0.5 to 5 dpf. Scale bars, (**E**) 50 µm, (**G**) 100 µm, (**H**) 5 µm. dpf, days post-fertilization; ALT, alanine aminotransferase; AST, aspartate aminotransferase.

Epidemiological research suggests that amoxicillin intake in infants and toddlers may predispose them to drug-induced liver injury [14]. In a clinical scenario, patients manifesting drug-induced liver injuries exhibit altered internal biochemical profiles, such as levels of triglycerides (TG) and total cholesterol (TC) and activities of alanine aminotransferase (ALT) and aspartate aminotransferase (AST). Our results showed no changes in these parameters in zebrafish larvae at 5 dpf after amoxicillin treatment (Figure 2A–D). Liver injuries are frequently coupled with lipid metabolic abnormalities. Employing Oil Red O staining, we scrutinized the liver for lipid accumulations. The results ruled out an increase of severe lipid deposition post-amoxicillin treatment (Figure 2E,F). Further investigations using WISH revealed stable expressions of liver function marker genes, *cp* and *abcb11b* (Figure 2G). This implied that amoxicillin did not detrimentally affect processes like liver copper transport and bile salt exportation. Histological examinations of the liver post-treatment discerned no pathological irregularities (Figure 2H). Collectively, our data suggest that amoxicillin treatment in zebrafish embryos is unlikely to culminate in drug-induced liver impairments.

### 2.3. Co-Treatment of Amoxicillin and Clavulanic Acid Affects Zebrafish Liver Development and Function

**Figure 3:**
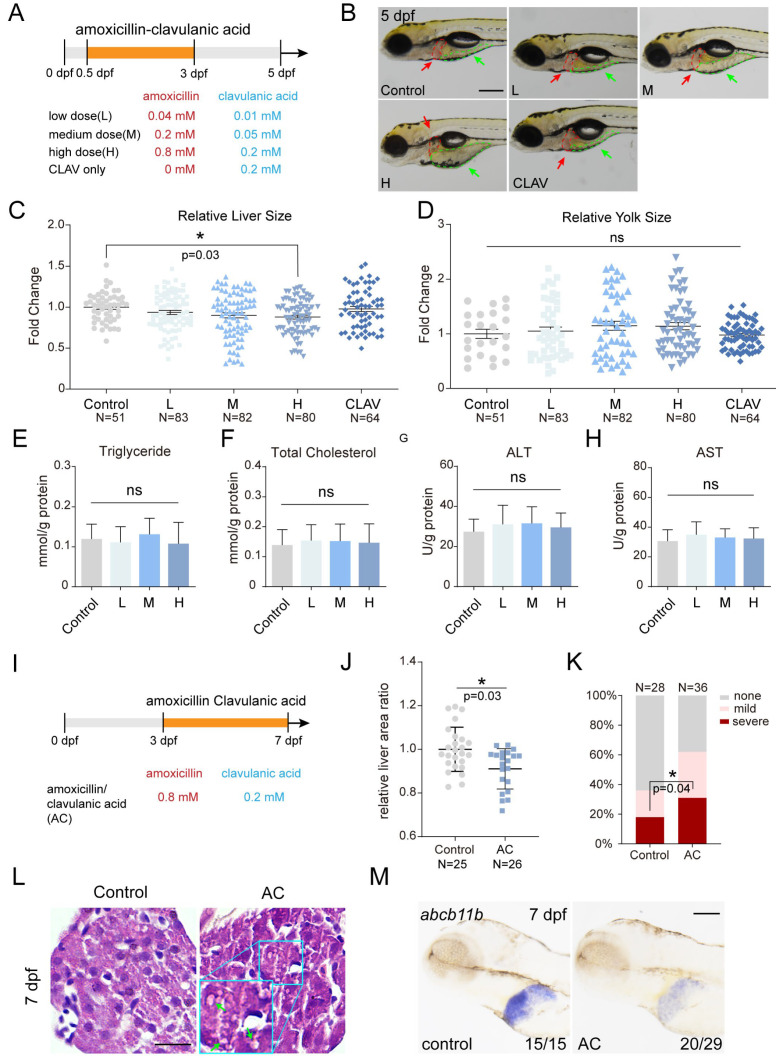
Co-treatment of amoxicillin and clavulanic acid affects zebrafish liver development and function. (**A**) Schematic diagram of early amoxicillin and clavulanic acid co-treatment from 0.5 to 3 dpf. (**B**) The liver and yolk morphology of *Tg(fabp10a:mCherry; ela:eGFP)* larvae at 5 dpf after early amoxicillin and clavulanic acid co-treatment. The red dashed lines and arrows indicate liver area; the green dashed lines and arrows indicate yolk area. (**C**,**D**) Quantification of relative sizes of liver and yolk in 5 dpf larvae after early amoxicillin and clavulanic acid co-treatment. The numbers of larvae analyzed in each group are indicated. Mean + s.e.m. In the ANOVA analysis, * indicates *p* < 0.05, ns—not significant. (**E**–**H**) Quantification of triglyceride, total cholesterol, ALT activity, and AST activity in 5 dpf larvae after early amoxicillin and clavulanic acid co-treatment. 3 independent experiments. Mean + s.e.m. In the ANOVA analysis, ns—not significant. (**I**) Schematic diagram of late amoxicillin and clavulanic acid co-treatment from 3 to 7 dpf. (**J**) Quantification of relative liver size in 7 dpf larvae after late amoxicillin and clavulanic acid co-treatment. The numbers of larvae analyzed in each group are indicated. Mean + s.e.m. In Student’s *t*-test, * indicates *p* < 0.05. (**K**) Quantification of hepatic steatosis by ORO staining in 7 dpf larvae after late amoxicillin and clavulanic acid co-treatment. The numbers of larvae analyzed in each group are indicated. In Chi-square test, * indicates *p* < 0.05. (**L**) H&E staining of liver tissues at 7 dpf after late amoxicillin and clavulanic acid co-treatment. Arrows point to fat vacuole. (**M**) WISH revealed the expression of *abcb11b* at 7dpf after late amoxicillin and clavulanic acid co-treatment. Numbers indicate the ratio of representative staining observed. Scale bars: (**B**,**L**) 100 µm, (**M**) 5 µm. dpf—days post-fertilization; AMX—amoxicillin; CLAV—clavulanic acid; AC—amoxicillin- clavulanic acid; L—low dose AC; M—medium dose AC; H—high dose AC.

Clinical practice often pairs amoxicillin with clavulanic acid to enhance antibacterial efficacy [9,17]. However, this combination has been linked to potential drug-induced liver injuries [39]. To study the potential developmental toxicity to zebrafish, we treated zebrafish embryos with a low, medium, or high dosage of the drug combination from 0.5 to 3 dpf (Figure 3A). The results revealed that the co-treatment with amoxicillin and clavulanic acid led to a reduction in liver size in a dose-dependent manner at 5 dpf, although treatment with amoxicillin or clavulanic acid alone had no effect at all, suggesting the potential impact of this drug combination on early liver development (Figure 3B,C and Figure S3A). It is pertinent to note that the larvae depend entirely on their internal yolk reserves for sustenance, essential for both movement and developmental processes, especially the liver’s metabolic functions. Larvae treated with amoxicillin and clavulanic acid exhibited a trend of enlarged yolk area compared to the control group, an indication of metabolic liver dysfunction (Figure 3B,D). The levels of TG and TC and activities of ALT and AST remained unchanged (Figure 3E–H).

Next, we administered a high dosage of amoxicillin and clavulanic acid from 3 to 7 dpf to observe their influence on the liver function of zebrafish larvae (Figure 3I). The liver size of the treated group was notably smaller than that of the control group at 7 dpf (Figure 3J). ORO staining revealed that 30% of the treated group exhibited severe lipid accumulation in the liver, representing a significant rise from 17% in the control group (Figure 3K). Furthermore, HE is staining showed increased presence of fatty vacuoles in the liver of the treated group (Figure 3L and Figure S3B). These observations imply that co-treatment with amoxicillin and clavulanic acid disrupts liver lipid metabolism. WISH revealed that expression of *abcb11b* was reduced in the treated group compared to the control group (Figure 3M), indicating compromise of the bile salt exportation process, which parallel clinical observations of cholestasis induced by combined use of amoxicillin and clavulanic acid. In summary, our results suggest that the combined use of amoxicillin and clavulanic acid leads to developmental toxicity in zebrafish liver and compromises liver function.

### 2.4. The Effect of Amoxicillin Pretreatment on Hepatic Steatosis Induced by a Fructose Diet

**Figure 4:**
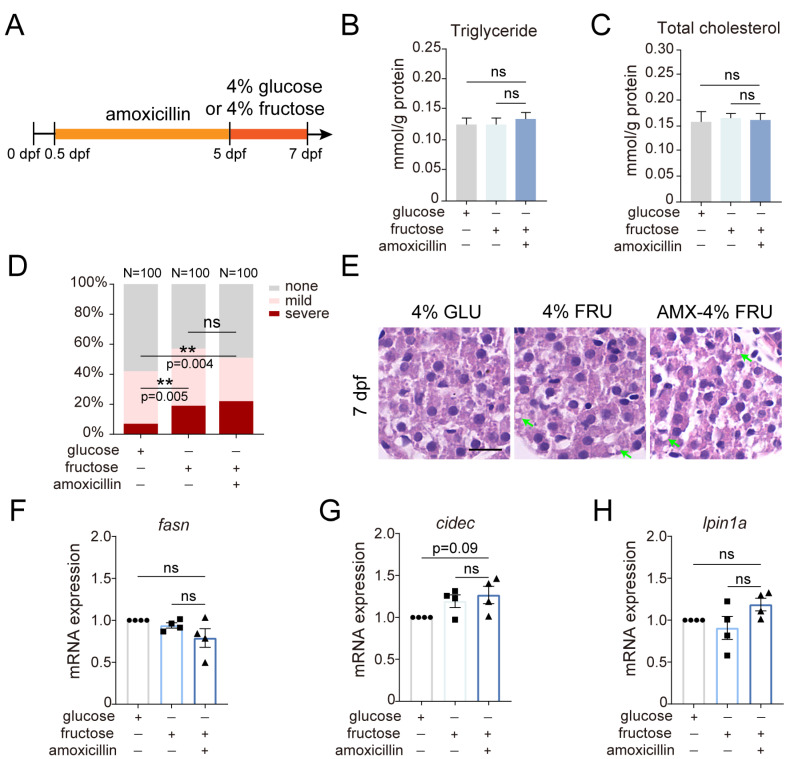
The effect of amoxicillin pretreatment on hepatic steatosis induced by short-term fructose diet. (**A**) Schematic diagram of pretreatment with amoxicillin alone followed by early short-term glucose or fructose diet. (**B**,**C**) Quantification of triglyceride and total cholesterol in 7 dpf larvae subject to short-term glucose or fructose diet with amoxicillin pretreatment. 3 independent experiments. Mean + s.e.m. In the ANOVA analysis, ns—not significant. (**D**) Quantification of hepatic steatosis by ORO staining in 7 dpf larvae. In the Chi-square test, ns—not significant, ** indicates *p* < 0.01. (**E**) H&E staining of liver tissues at 7 dpf. Arrows point to fat vacuoles. Scale bar: 5 µm. (**F**–**H**) Quantification of the expression of *fasn*, *cidec*, and *lpin1a* at 7 dpf by real-time PCR. Four independent experiments. Mean + s.e.m. In the ANOVA analysis, ns—not significant. dpf—days post-fertilization; AMX—amoxicillin; GLU—glucose; FRU—fructose.

Considering the extensive utilization of amoxicillin during pregnancy and its impact on neonates [14,15], our investigation was expanded to determine if embryonic exposure to amoxicillin alters disease susceptibility of the offspring. Researchers have linked fructose consumption directly to the development and progression of NAFLD. We employed a previously reported zebrafish NAFLD model induced by a fructose diet [40]. Amoxicillin pretreatment was carried out from 0.5 to 5 dpf, followed by a 4% glucose diet or a 4% fructose diet from 5 to 7 dpf (Figure 4A). The amoxicillin-pretreated group showed no significant differences in TG and TC levels compared to the fructose group without amoxicillin pretreatment (Figure 4B,C). The percentages of severe lipid deposition by ORO staining increased in the fructose group (19%) compared to the glucose group (7%), but was not further elevated in the amoxicillin-pretreated group (22%) (Figure 4D). HE staining also revealed no further increase in lipid vacuoles, suggesting that amoxicillin pretreatment did not intensify fructose-induced hepatic steatosis (Figure 4E). Additionally, we analyzed the expression of several genes related to lipid metabolism. The slight upregulation of cell death inducing DFFA like effector c (*cidec*) and *lipin 1a* (*lpin1a*) suggests that amoxicillin pretreatment may affect lipid metabolism (Figure 4F–H).

**Figure 5:**
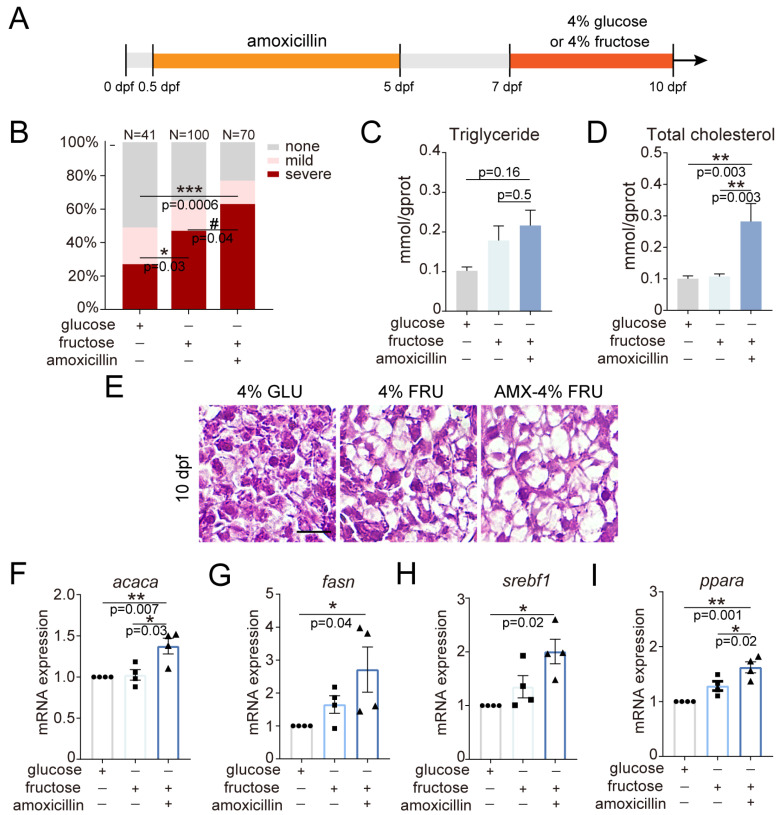
The effect of amoxicillin pretreatment on hepatic steatosis induced by long-term fructose diet. (**A**) Schematic diagram of pretreatment with amoxicillin alone followed by later long-term glucose or fructose diet. (**B**) Quantification of hepatic steatosis by ORO staining in 10 dpf larvae subject to long-term glucose or fructose diet with amoxicillin pretreatment. The numbers of larvae analyzed in each group are indicated. In the Chi-square test, * and *** indicate *p* < 0.05, *p* < 0.001, and # *p* < 0.05, respectively. (**C**,**D**) Quantification of triglyceride and total cholesterol in 10 dpf larvae. 3 independent experiments. Mean + s.e.m. In the ANOVA analysis, ** indicates *p* < 0.01 (**E**) H&E staining of liver tissues at 10 dpf. Scale bar: 5 µm. (**F**–**I**) Quantification of the expression of *pparα*, *acaca*, *fasn*, and *srebf1* at 10 dpf by real-time PCR. Four independent experiments. Mean + s.e.m. In the ANOVA analysis, * and ** indicate *p* < 0.05 and *p* < 0.01, respectively. dpf—days post-fertilization; AMX—amoxicillin; GLU—glucose; FRU—fructose.

To discern whether amoxicillin exposure during embryonic stages plays a role in the development of NAFLD, we refined the diet-induced NAFLD model by adjusting the treatment time of fructose or glucose to 7 to 10 dpf, which did not affect the liver sizes (Figure 5A and Figure S3C). ORO staining revealed that the percentages of severe hepatic steatosis increased in the fructose group (47%) compared to the glucose group (27%) and further increased in the amoxicillin-pretreated group (63%) (Figure 5B). While TG level showed a minor increase, TC level was significantly elevated in the amoxicillin-pretreated group (Figure 5C,D). HE staining indicated pronounced morphological changes in the liver cells of the amoxicillin-pretreated group: hepatocytes appeared swollen and contained large lipid vacuoles (Figure 5E). We then analyzed the expression of several genes that play a crucial role in lipid metabolism. The expression levels of acetyl-CoA carboxylase alpha (*acaca*), fatty acid synthase (*fasn*), and sterol regulatory element-binding transcription factor 1 (*srebf1*) significantly increased in the amoxicillin-pretreated group (Figure 5F–H). Interestingly, expression of peroxisome proliferator-activated receptor alpha (*ppara*) also increased in the amoxicillin-pretreated group (Figure 5I). These data indicate that embryonic amoxicillin exposure can exacerbate fructose-induced NAFLD.

## 3. Discussion

Amoxicillin, a semi-synthetic penicillin, has been extensively utilized in clinical practice since the 1970s [9]. The prevailing view is that amoxicillin is safe for treating bacterial infections, which has led to a higher uptake of amoxicillin treatment among pregnant women [41]. Therefore, the impact of amoxicillin usage during pregnancy on multi-organ development and related diseases in offspring needs further exploration. We have developed a zebrafish model to investigate the effects of amoxicillin exposure during embryonic stages on liver development and disease susceptibility later in life.

We initiated amoxicillin treatment at 12 hpf, which corresponds to the late gestational phase in humans, to circumvent the teratogenic potential of amoxicillin. Previous studies have reported that exposure to 0.6 mM amoxicillin from the zygote stage resulted in higher occurrences of malformations in embryos, characterized by edemas and tail deformities [37]. This concentration after conversion is equivalent to the daily oral dosage pregnant women receive but is higher than clinical blood concentrations; the exact differences still await experimental determination due to the lack of data for drug permeability through the zebrafish chorion. Moreover, the impact of amoxicillin on the development of vital organs such as the liver and heart remains unexplored. Zebrafish liver development mainly comprises three stages: specification, budding/differentiation, and outgrowth [42]. Hepatic specification in zebrafish begins early in development after the formation of the foregut endoderm at 1 dpf [43]. Many transcription factors (such as GATA6, Hhex, and Foxa3) are implicated in liver bud formation and hepatoblast specification [44,45,46]. We found no change in the expression of these genes following amoxicillin exposure, suggesting that amoxicillin does not impact zebrafish liver function by altering early liver development.

Next, we focused on whether amoxicillin exposure causes a decline in liver function in zebrafish at 5 dpf, a stage when liver metabolic functions mature [47]. This aligns with a previous report that unlike acetaminophen, which led to a significant reduction in liver area, amoxicillin did not cause severe liver injury [48]. The biochemical results of ALT and AST activities and the expression of hepatic function markers *cp* and *abcb11b* indicated that amoxicillin treatment did not cause hepatic functional changes. The gene *cp*, which encodes a multi-copper oxidase that plays a critical role in iron metabolism [49], often serves as a biomarker for assessing the function of liver cells in zebrafish [50]. The bile salt export pump ABCB11/BSEP is a liver-specific ATP-binding cassette transporter that mediates the biliary excretion of bile salts from hepatic cells [51]. Liver injury inevitably compromises liver function, which in turn affects hepatic lipid metabolism. The biochemical results of TG and TC levels and ORO staining indicated no impact on zebrafish lipid metabolism attributable to amoxicillin. HE staining did not reveal large lipid droplets. These results suggest that amoxicillin exposure does not affect liver development and function.

Amoxicillin, which is unable to inhibit β-lactamase-producing bacteria, is often used in conjunction with clavulanic acid clinically. However, their combined use can lead to drug-induced liver injury [9]. The co-treatment of amoxicillin and clavulanic acid reduced liver area during early liver development in a dose-dependent manner with no significant alterations observed in the yolk area and biochemical indicators, suggesting an impact on liver proliferation rather than function. Zebrafish liver completes morphogenesis by 3 dpf and establishes basic functionality by 5 dpf [52]. Late treatment with amoxicillin and clavulanic acid significantly reduced liver area. The decreased expression of *abcb11b* indicated liver function impairment. The increase in ORO staining and the augmentation of lipid droplets in HE further indicated a disorder in liver lipid metabolism. Using the zebrafish model, we confirmed that concurrent use of amoxicillin and clavulanic acid results in bile salt excretion disruption [53], which might be interconnected with the observed effects on lipid metabolism.

The Developmental Origins of Health and Disease (DOHaD) theory suggests that individuals subjected to adverse events during development may have an increased risk of non-communicable diseases in adulthood [54]. The susceptibility of offspring to diseases due to prenatal drug exposure has been attracting attention [55]. The safety of amoxicillin, which is widely used during pregnancy for treating infections, needs further exploration. Previous studies have demonstrated that early life exposure to antibiotics in HFD mouse models alters adult metabolic levels [26]. However, while the HFD is a widely used model for studying NAFLD, the relationship between early antibiotic exposure and NAFLD remains unexplored. Researchers have linked fructose consumption directly to the development and progression of NAFLD. The consumption of calorie-dense foods, especially those with added fructose, contributes to the increasing prevalence of NAFLD [56]. Patients with NAFLD show higher fructose consumption and increased expression of the fructokinase gene compared to patients without NAFLD [57]. Therefore, we use the FD model of NAFLD to study the relationship between early amoxicillin exposure and subsequent development of NAFLD.

We first utilized a previously established short-term fructose-induced zebrafish NAFLD model [34,40] and validated that FD increased both the extent of severe ORO staining and the number of lipid droplets as seen in HE staining. However, exposure to amoxicillin during the embryonic stage did not exacerbate NAFLD. We then employed an improved long-term fructose-induced NAFLD model by extending the time of FD. Amoxicillin pretreatment significantly increased the proportion of severe ORO staining and the level of TC, indicating the occurrence of NAFLD. Fasn and Srebf1 are crucial nuclear transcription factors which maintain the homeostasis of hepatic lipid metabolism [58,59], and overexpression of Srebf1 could activate the synthesis of fatty acids and triglycerides [60]. Following amoxicillin pretreatment and long-term fructose diet, a significant upregulation of *srebf1* and *fasn* was observed, suggesting that enhanced lipid synthesis might be a key factor leading to more severe NAFLD. PPARs are members of the nuclear receptor superfamily and play a vital role in the regulation of physiological functions including adipocyte differentiation and lipid metabolism regulation [61]. PPARα is the predominant type expressed in the liver [62]. Expression of the *pparα* gene was upregulated in the amoxicillin-pretreated group. The activation of hepatic PPAR signaling could serve as a compensatory mechanism for lipid accumulation. Recent epidemiological studies also suggest that children exposed to antibiotics early in life have an increased risk of obesity [63,64]. Our results are in line with these studies and elevates serious concerns about the risk of developing NAFLD, particularly with the rising prevalence of high-sugar, high-calorie diets, which lead to earlier onset of the disease. Consequently, developing animal models to understand the effects of antibiotics on NAFLD and other diseases becomes crucial.

In summary, our findings demonstrate that amoxicillin treatment alone does not affect liver development and function in zebrafish. However, when used in combination with clavulanic acid, it induces liver injury, which is consistent with current clinical observations. Exposure to amoxicillin exacerbates NAFLD phenotypes, a phenomenon driven by the upregulation of lipid synthase *srebf1* and *acaca*. The PPAR signaling pathway may play a role in the dysregulation of lipid homeostasis. This study sheds more light on our understanding of amoxicillin-induced susceptibility to diseases in offspring.

## 4. Methods and Materials

### 4.1. Zebrafish

Zebrafish were raised and maintained under standard conditions. All experiments were performed according to institutional and national animal welfare guidelines and were approved by the Institutional Animal Care and Use Committee of Wuhan University Center for Animal Experiment (WQ20210083, 2021-3-3). The zebrafish lines used in this study included wild-type AB strain and transgenic lines *Tg(vmhc:mCherry-NTR)* and *Tg(fabp10a:mCherry; ela:eGFP)*. Embryos over 24 hpf were maintained in E3 water with 0.003% PTU (1-phenyl-2-thiourea, Sigma, St. Louis, MO, USA) to prevent pigmentation.

### 4.2. Chemical Treatment

Amoxicillin and clavulanic acid were obtained from Meilunbio (Dalian, China). Glucose and fructose were obtained from Solarbio (Beijing, China). Stock solutions were prepared, and concentration gradients were diluted in E3 water with 0.003% PTU. The embryos at 12 hpf were distributed into 6-well plates (30 embryos/well) in 5 mL drug solution. The drug solution was replaced every 24 h, and dead embryos were removed to prevent deterioration of the solution.

### 4.3. Quantification of Liver Area

After being anesthetized with 1 mg/mL tricaine (3-aminobenzoic acid ethyl ester, Sigma), the larvae were fixed in 1% low melting agarose gel with left side upwards. Liver images were obtained with a Nikon SMZ25 fluorescence stereo microscope. The liver areas were measured using ImageJ software (version 1.53t).

### 4.4. Cardiac Function Analysis

Movies of beating hearts from embryos at 3 dpf were recorded using a Nikon SMZ25 stereo microscope equipped with a Nikon DS-Fi3 camera. Images from movies were then used to measure the lengths of long axis (a) and short axis (b) between the myocardial borders of ventricles at diastole and systole, respectively. The fractional shortening (FS) was calculated using the formula FS = (length at diastole − length at systole)/(length at diastole) × 100. End-systolic or end-diastolic volumes were calculated using the formula volume = 4/3 πab^2^. To measure the heart rate, the number of sequential heart contractions in a 15 s interval was counted.

### 4.5. Biochemical Assay

ALT activity assay kit, AST activity assay kit, LDL assay kit, total cholesterol assay kit, and triglyceride assay kit were purchased from Nanjing Jiancheng Bioengineering Institute (Nanjing, China). Fifty larvae were homogenized in saline solution for one test following the manufacturer’s instructions. The protein concentrations were quantified by using a BCA protein assay kit (PC0020, Solarbio).

### 4.6. Whole-Mount Oil Red O Staining

Oil red O (O0625, Sigma) staining was performed at 7 dpf. Zebrafish larvae were fixed in 4% paraformaldehyde (PFA) overnight at 4 °C and washed three times with 1× PBS for 5 min each. The fixed larvae were infiltrated with PBS solution containing 25%, 50%, 75%, or 100% 1,2-propylene glycol 5 min respectively then stained with 0.5% oil red O in 100% 1,2-propylene glycol in the dark for 2 h at room temperature. Images were obtained with a Nikon SMZ25 stereo microscope (Nikon Corporation, Tokyo, Japan).

### 4.7. Quantitative Real-Time PCR

Total RNA from thirty larvae were extracted at the indicated stages using the RNAiso Plus reagent (TaKaRa Bio, Beijing, China) following the manufacturer’s instruction. cDNA was synthesized with an Evo M-MLV kit (ACCURATE BIOTECHNOLOGY (HUNAN) Co., Ltd., ChangSha, China). cDNA was diluted 1:4 with nuclease-free water and used for qPCR reactions. The gene expression profile was carried out in triplicate with Taq Pro Universal SYBR qPCR Master Mix (Vazyme Biotech Co., Ltd., Nanjing, China) and normalized by β-actin as internal control. The sequences of primers used are listed in Table S1.

### 4.8. Whole-Mount In Situ Hybridization

WISH was performed using digoxigenin-labeled antisense RNA probes. Briefly, zebrafish embryos were fixed in 4% paraformaldehyde (PFA) overnight at 4 °C. After being rinsed with PBS, embryos were digested with 20 μg/mL proteinase K in PBST (0.1% Tween20 in PBS) and re-fixed with 4% PFA for 20 min; this was followed by pre-hybridization in hybridization buffer for 3 to 4 h at 68 °C and incubation overnight with probes (1 ng/μL) diluted in hybridization buffer at 68 °C. Detection was performed using 1:4000 dilution of anti-Digoxigenin-AP antibody and visualized by NBT/BCIP substrate reaction. To generate antisense riboprobes, transcription templates were amplified from cDNA and subcloned into pGEM-T Easy vector (Vazyme Biotech Co., Ltd., Nanjing, China). Linearized constructs were transcribed in vitro using T7 RNA polymerase (Thermo Fisher Scientific, Waltham, MA, USA) with DIG RNA Labeling Mix (Roche, Basel, Switzerland).

### 4.9. Hematoxylin-Eosin Staining

Hematoxylin-eosin staining was performed at the indicated stages. The protocol for HE staining was described previously [65]. The fixed larvae were stained with hematoxylin and eosin. Images were obtained with a Leica DM1000microscope (Wetzlar, Germany).

### 4.10. Statistical Analyses

Statistical analyses were performed using GraphPad Prism 8.0 software (GraphPad 8.0.2, La Jolla, CA, USA). Statistical significance was defined as a threshold of *p* < 0.05 determined by Student’s t-test between two groups, ANOVA analysis between more than two groups, or chi-square test in quantification of the percentage of severe ORO staining.

## Data Availability

All data generated or analyzed during this study are included in this published article and its Supplementary Materials.

## Supplementary Materials

The following supporting information can be downloaded at: https://www.mdpi.com/article/10.3390/ijms25052744/s1.
