# Supplementary material for: Embryonic Amoxicillin Exposure Has Limited Impact on Liver Development but Increases Susceptibility to NAFLD in Zebrafish Larvae
Source: Int J Mol Sci. 2024 Feb 27;25(5):2744. doi: 10.3390/ijms25052744 (PMC10931932; doi:10.3390/ijms25052744)
Supplement: Supplementary file 1 [file ijms-25-02744-s001.zip › ijms-2862819-supplementary.pdf]

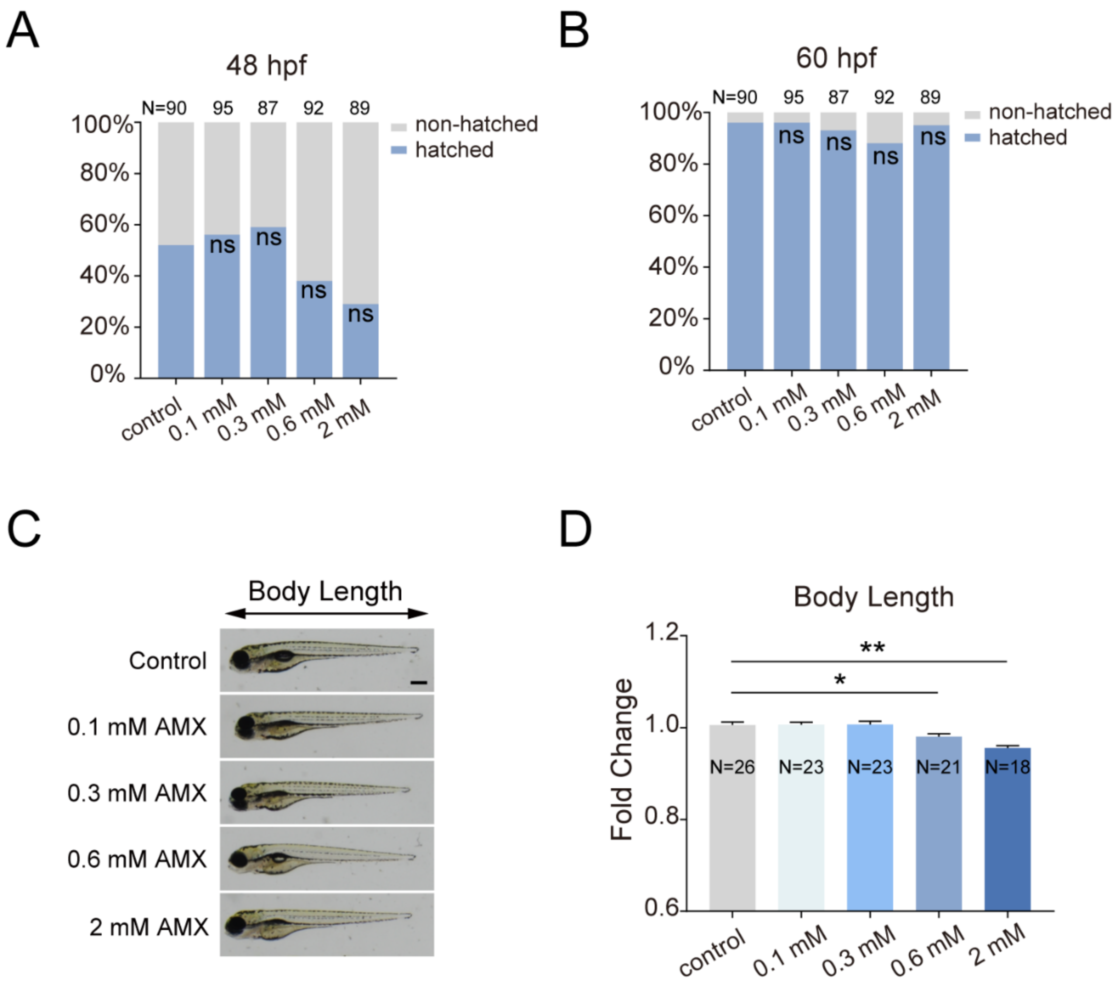

Supplementary figure 1. Effects of amoxicillin treatment on zebrafish larval development

(A, B) Quantification of hatching rate at 48 and 60 hpf with amoxicillin treatment. The numbers of embryos analyzed in each group are indicated. Chi-square test, ns, not significant.

(C, D) Quantification of the relative body length of 4 dpf larvae with amoxicillin treatment. Scale bar, 100 μm. The numbers of embryos analyzed in each group are indicated. Mean + s.e.m. ANOVA analysis, *$P < 0.05$, **$P < 0.01$.

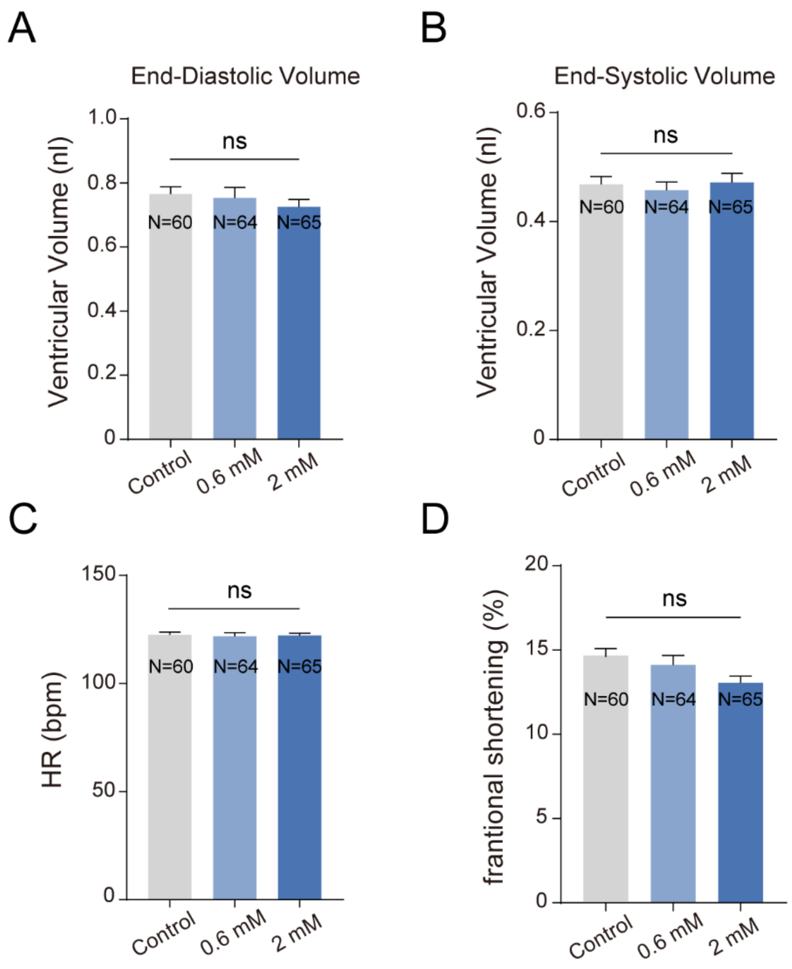

Supplementary figure 2. Effects of amoxicillin treatment on zebrafish heart development and function

(A, B) Quantification of the ventricular end-diastolic volume and end-systolic volume in 3 dpf embryos with amoxicillin treatment. The numbers of embryos analyzed in each group are indicated. Mean + s.e.m. ANOVA analysis, ns, not significant.

(C) Quantification of heart rates in 3 dpf embryos with amoxicillin treatment. The numbers of embryos analyzed in each group are indicated. Mean + s.e.m. ANOVA analysis, ns, not significant.

(D) Quantification of ventricular fractional shortening in 3 dpf embryos with amoxicillin treatment. The numbers of embryos analyzed in each group are indicated. Mean + s.e.m. ANOVA analysis, ns, not significant.

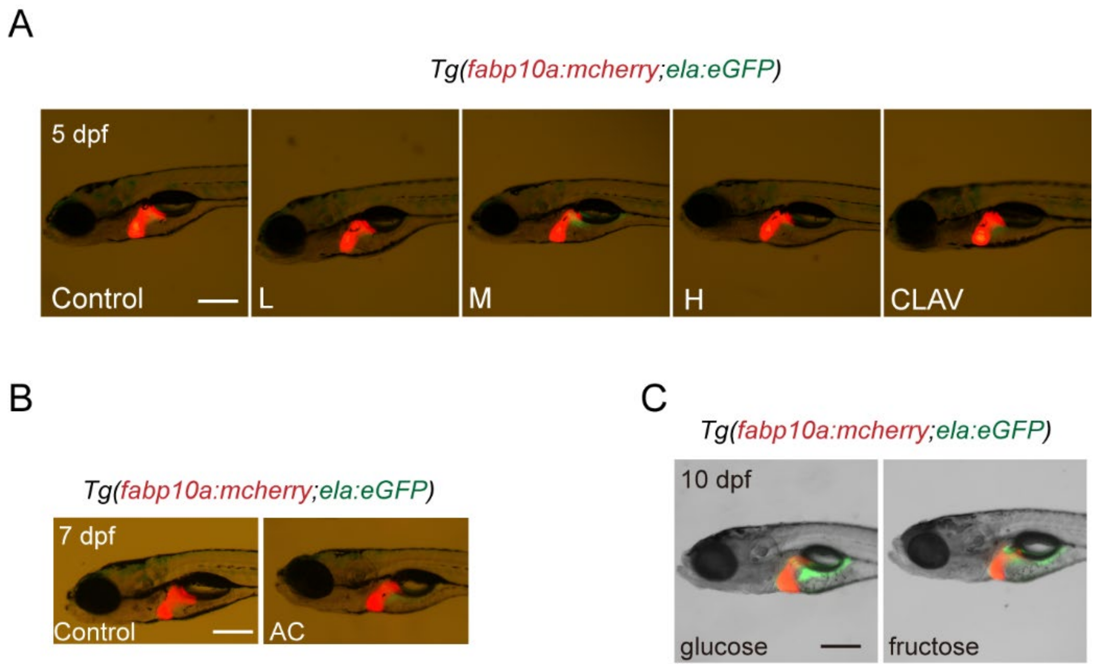

Supplementary figure 3. Fluorescence of the liver following treatment with amoxicillin-clavulanate acid or fructose

(A) Liver (red) size at 5 dpf after amoxicillin and clavulanic acid co-treatment as shown in the transgenic reporter line *Tg(fabp10a:mCherry; ela:eGFP)*. Lateral view, anterior to the left.

(B) Liver (red) size at 7 dpf after amoxicillin and clavulanic acid co-treatment.

(C) Liver (red) size at 10 dpf after glucose/fructose treatment.

Scale bars, 100 µm. AC, amoxicillin- clavulanic acid; L, low dose AC; M, medium dose AC; H, high dose AC; CLAV, clavulanic acid.

Table S1. The primer sequence for RT-PCR.

Gene	Forward primer (5'-3')	Reverse primer (5'-3')
actb1	CAGCCTTCCTTCCTGGGTAT	GCCATACAGAGCAGAAGCCA
acaca	CCCCAGAGTTGAGTGTGTCT	TCCTCTTCACCGTTTCCTCC
ppara	TGCTGGACTACCAGAACTGTGACA	TGCTGGCTGAGAACACTTCTGAG
fasn	GGAGCAGGCTGCCTCTGTGC	TTGCGGCCTGTCCCACTCCT
srebf1	CATCCACATGGCTCTGAGTG	CTCATCCACAAAGAAGCGGT
cidec	TGTGCTCTCAACATCCCTGTC	ATCACCTTGTGGTGCAGGTC
lpin1a	GAAGCGTGAGGACAATGGAGAC	ACATTGCTCTGCTGCCTGAAG
